# Training of paced breathing at 0.1 Hz improves CO_2_ homeostasis and relaxation during a paced breathing task

**DOI:** 10.1371/journal.pone.0218550

**Published:** 2019-06-20

**Authors:** Mikołaj Tytus Szulczewski

**Affiliations:** Faculty of Psychology, University of Warsaw, Warsaw, Poland; Universidade Federal de Juiz de Fora, BRAZIL

## Abstract

Volitional control of breathing often leads to excessive ventilation (hyperventilation) among untrained individuals, which disrupts CO_2_ homeostasis and may elicit a set of undesirable symptoms. The present study investigated whether seven days of training without any anti-hyperventilation instructions improves CO_2_ homeostasis during paced breathing at a frequency of 0.1 Hz (6 breaths/minute). Furthermore, the present study investigated the effects of training on breathing-related changes in affective state to examine the hypothesis that training improves the influence of slow paced breathing on affect. A total of 16 participants performed ten minutes of paced breathing every day for seven days. Partial pressure of end-tidal CO_2_ (PetCO_2_), symptoms of hyperventilation, affective state (before and after breathing), and pleasantness of the task were measured on the first, fourth, and seventh days of training. Results showed that the drop in PetCO_2_ significantly decreased with training and none of the participants experienced a drop in PetCO_2_ below 30 mmHg by day seven of training (except one participant who already had PetCO_2_ below 30 mmHg during baseline), in comparison to 37.5% of participants on the first day. Paced breathing produced hyperventilation symptoms of mild intensity which did not decrease with training. This suggests that some participants still experienced a drop of PetCO_2_ that was deep enough to produce noticeable symptoms. Affective state was shifted towards calmness and relaxation during the second and third laboratory measurements, but not during the first measurement. Additionally, the breathing task was perceived as more pleasant during subsequent laboratory measurements. The obtained results showed that training paced breathing at 0.1 Hz led to decrease in hyperventilation. Furthermore, the present study suggests that training paced breathing is necessary to make the task more pleasant and relaxing.

## Introduction

In recent decades, slow paced breathing (most often around the 0.1 Hz frequency) has been widely studied as a method for improving cardiovascular health and decreasing affective arousal. Breathing at frequencies around 0.1 Hz is used in biofeedback-based methods aimed at increasing heart rate variability [1, 2, 3]. Research has shown promising results regarding the effectiveness of heart rate variability biofeedback in treating depression [4, 5], anxiety [6], asthma [7], fibromyalgia [8], posttraumatic stress disorder [9, 10], as well as other conditions. Breathing at lower frequencies has also been successfully tested as a method to improve baroreflex function in the complementary treatment of cardiovascular diseases [11, 12, 13]. Furthermore, research suggests that breathing at 0.1 Hz decreases pain, which makes it a promising complementary method for the treatment of chronic pain [14, 15, 16]. It can also improve sleep quality [17, 18] and decrease affective arousal [19], so it is a widely-used method of emotion regulation, that has recently been implemented in multiple mobile applications [20, 21].

Spontaneous breathing is primarily controlled in an automatic manner by respiratory networks in the medulla oblongata and pons on the basis of information from CO_2_, O_2_, and pH chemoreceptors and respiratory mechanoreceptors [22] with descending influences related to emotional and cognitive processes [23]. During paced breathing, volitional breathing shifts the balance in neural control of breathing toward higher brain centers instead of the control mechanism in the brainstem [24, 25]. Furthermore, paced breathing prevents the use of compensatory mechanisms such as apnea. As a result, some untrained participants tend to hyperventilate during slow paced breathing [26, 27, 28]. Hyperventilation leads to a decrease in arterial pressure of CO_2_, and when this decrease is large enough it causes several undesirable physiological and psychological changes, such as increase in heart rate, paresthesia and tetany, dizziness, lightheadedness, and increased emotional arousal [29, 30, 31, 32]. For this reason, hyperventilation is considered undesirable during paced breathing exercises.

The effects of paced breathing training on hyperventilation during paced breathing remains largely unknown. One study on nine weeks of slow paced breathing training for hot flashes reported adverse effects such as dizziness, palpitations, and tingling in extremities, which are typical symptoms of hyperventilation [33]. This study suggests sustained hyperventilation during paced breathing training. Some studies on paced breathing training implemented various methods to counteract hyperventilation and successfully taught participants to avoid hyperventilation One study on heart rate variability suggests that daily paced breathing training removes hyperventilation [2]. In this study, participants stopped hyperventilating after three days of training. However, capnography was used during training sessions and researchers instructed participants to breathe shallower whenever the partial pressure of end-tidal CO_2_ (PetCO_2_) dropped too much. Furthermore, multiple studies on the use of breathing training in panic disorder and asthma have shown that capnometry biofeedback can be successfully used to decrease hyperventilation during paced breathing [34, 35, 36, 37]. However, often neither capnography measurement nor an anti-hyperventilation instruction is used in paced breathing training [17, 33, 38]. Furthermore, paced breathing is widely used in mobile applications [21, 22], where the issue of potential hyperventilation is frequently overlooked. Therefore, it is important to investigate the effects of training on CO_2_ homeostasis during paced breathing tasks without any anti-hyperventilation instruction. It is possible that the mere training of paced breathing tasks improves CO_2_ homeostasis during paced breathing.

Change in affective state is another effect of paced breathing which can be modified by training. More specifically, the effects of slow paced breathing on affective state may improve with training. Previous studies that investigated the influence of paced breathing at frequencies around 0.1 Hz on affect among untrained participants provided mixed results: some reported relaxing and anti-anxiety effects [39, 40, 41, 42, 43] and others did not [44, 45, 46, 27, 28]. At the same time, a lot of studies suggest that regular practice of paced breathing at frequencies around 0.1 Hz improves emotional functioning, for example by reducing anxiety (for meta-analysis see: [6]). This inconsistency suggests that paced breathing tasks may require training to produce relaxing effects.

The present study aimed to investigate how daily practice of breathing at 0.1 Hz for seven days influences arterial CO_2_ homeostasis, hyperventilation symptoms, breathing-related changes in affective state, and the pleasantness of the task. No instructions aimed at influencing the depth of breathing were used in this study. The primary question was whether training decreases the tendency to hyperventilate. The secondary question was whether training slow paced breathing improves its effects on affective state. It was expected that, after training, hyperventilation and symptoms of hyperventilation would decrease and paced breathing would be perceived as more pleasurable and would decrease affective arousal more effectively than during the first performance of the task.

## Method

### Participants

A total of 16 participants were recruited via social media (8 men and 8 women). Participants were 19 to 29 years old (*M* = 22.37, *SD* = 3.32). Exclusion criteria based on self-report were chronic respiratory diseases (e.g. asthma), other chronic diseases (e.g. diabetes), cardiovascular diseases, psychiatric and neurological conditions, and any condition that may affect the ability to breathe through one’s nose (e.g. allergy). Participants were debriefed about their health by the experimenter upon arrival at the laboratory. Furthermore, only participants who had not participated in any form of breathing training, such as yoga or singing classes with breathing exercises, were recruited. Participants were asked to refrain from drinking beverages with caffeine and smoking tobacco for four hours before laboratory measurements. Participants were paid the equivalent of 23 euros for participating in the study. Approval from the local ethics committee was obtained (the ethical committee of the University of Warsaw).

### Apparatus and materials

Measurement of affective state was conducted with the use of the Polish translation of the Two-Dimensional Mood Scale [47]. This questionnaire is based on the two dimensional model of affect [48, 49]. Four measures of affective state were computed: arousal and valence, and vitality and stability. The latter two are the arousal and valence axes rotated by 45°. The rotated axes are also known as pleasant and unpleasant arousal, and because these terms are more common they will be used in the rest of the text [50, 51]. Symptoms of hyperventilation were measured on a 0–6 Likert scale which ranged from 0 (*not experiencing the symptom at all*) to 6 (*experiencing the symptom with maximal imaginable intensity*). Symptoms of hyperventilation were drawn from the Nijmegen Questionnaire [52] and a study which measured hyperventilation symptoms [29]. The following hyperventilation symptoms were examined: dizziness, tingling and pricking, numbness, headache, and increased muscle tension in hands and feet. Means of pre- and post-task symptoms were computed for further analysis. Furthermore, participants were asked to rate how pleasant they found the task on a scale which ranged from 0–6 (from *extremely unpleasant* to *extremely pleasant*).

Respiratory rate and PetCO_2_ were measured with a Capnocheck Sleep Capnograph 9004 (BCI). Before the experiment, the capnograph was calibrated with 5% CO_2_ calibration gas. Data were transferred to a PC computer and the mean value of PetCO_2_ and respiratory rate were computed in MATLAB (Release 7.1, TheMathWorks, Natick, MA).

### Procedure

The study lasted seven days. Participants performed a ten-minute-long paced breathing task every day. Laboratory measurements of PetCO_2_ were conducted on the first, fourth, and seventh days of practise. On the other days, participants used an online procedure. They logged-in using individual codes to access the experimental procedure. To ensure that the participants performed the task at home, the experimenter had access to the system and could examine who had logged in and when. Four participants skipped one day of training.

Laboratory measurements were conducted in a soundproof room with stable temperature. The experimenter sat in a separate room and monitored the experimental procedure and capnogram. The experimental procedure was automated and ran on OpenSesame 3.0.7 software [53]. The experiment begin with a six-minute resting period during which participants were asked to sit comfortably with opened eyes. The first minute was an adaptation period and the next five minutes were the baseline period which was used in analysis. Then, participants answered questions about current affective state (Two-Dimensional Mood Scale) and symptoms of hyperventilation. Next, participants breathed for ten minutes at a frequency of 0.1 Hz. Breathing was paced by an acoustic signal with different pitches for inhalation and exhalation and changing volume (it was loudest in the middle of each breathing phase). Furthermore, the words “inhale” and “exhale” were displayed on the screen. Inhalation lasted four seconds and exhalation lasted six seconds, a ratio that resembles the spontaneous ratio during breathing at 0.1 Hz when only the length of the whole breathing cycle is paced [54]. After the breathing task, participants once more answered the questions about affective state as well as questions about symptoms of hyperventilation; they also rated how pleasant they found the task. Home practice was done using the same audiovisual pacer. During home practice, participants were asked to sit down comfortably with opened eyes.

### Statistical analysis

Statistical analyses were conducted in IBM SPSS Statistics for Windows (version 22.0, IBM Corp., Armonk, NY) with significance set at *p* < 0.05. ANOVAs for repeated measures were conducted to examine the effects of day of laboratory measurement (first x second x third laboratory session) and phase of the experiment (baseline x breathing at 0.1 Hz) on respiratory rate, PetCO_2_, hyperventilation symptoms, and scores on four dimensions of affective state. A repeated measures ANOVA was conducted to examine the effects of day of laboratory measurement (first x second x third laboratory session) on the pleasantness of the task. When the sphericity assumption was violated, Greenhouse-Geisser correction was used. Post-hoc analyses were conducted with Bonferroni correction. When the interactions in the ANOVAs were significant, paired sample *t*-tests were computed for differences between baseline and measurement during the breathing task for PetCO_2_ and post-task measurements of self-reported variables. Linear regression was performed with changes in symptoms of hyperventilation between baseline and paced breathing as a dependent variable and PetCO_2_ during paced breathing as predictor. Confidence intervals for the plots were calculated with the use of the data normalization procedure proposed by Morey [55].

## Results

**Fig 1:**
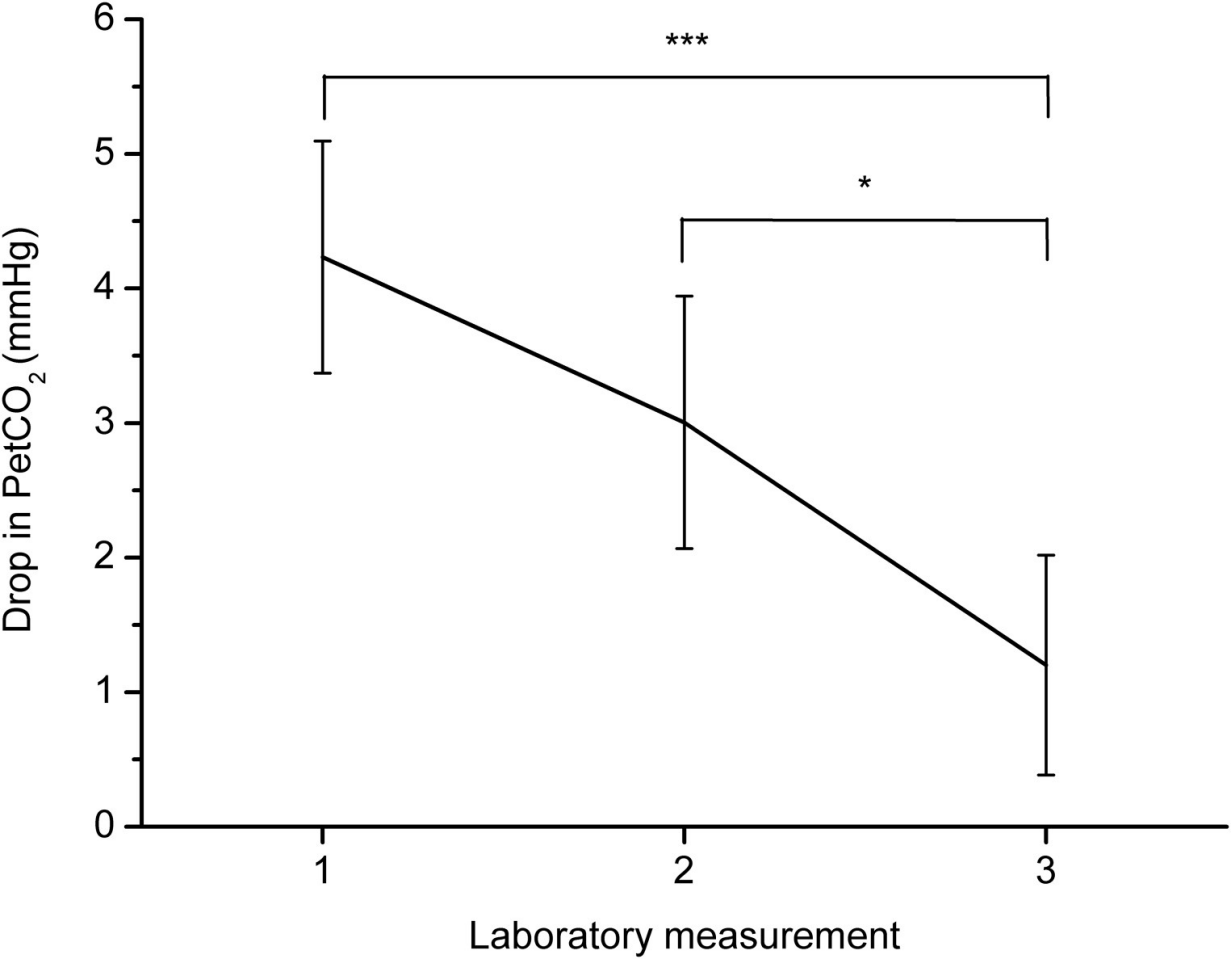
Changes in drop in PetCO_2_ between the three laboratory measurements with 95% confidence intervals. Changes computed by subtraction of PetCO_2_ during paced breathing from PetCO_2_ during baseline. Note. * *p* < .05 *** *p* < .001; the significance of the difference between the laboratory measurements is indicated.

**Fig 2:**
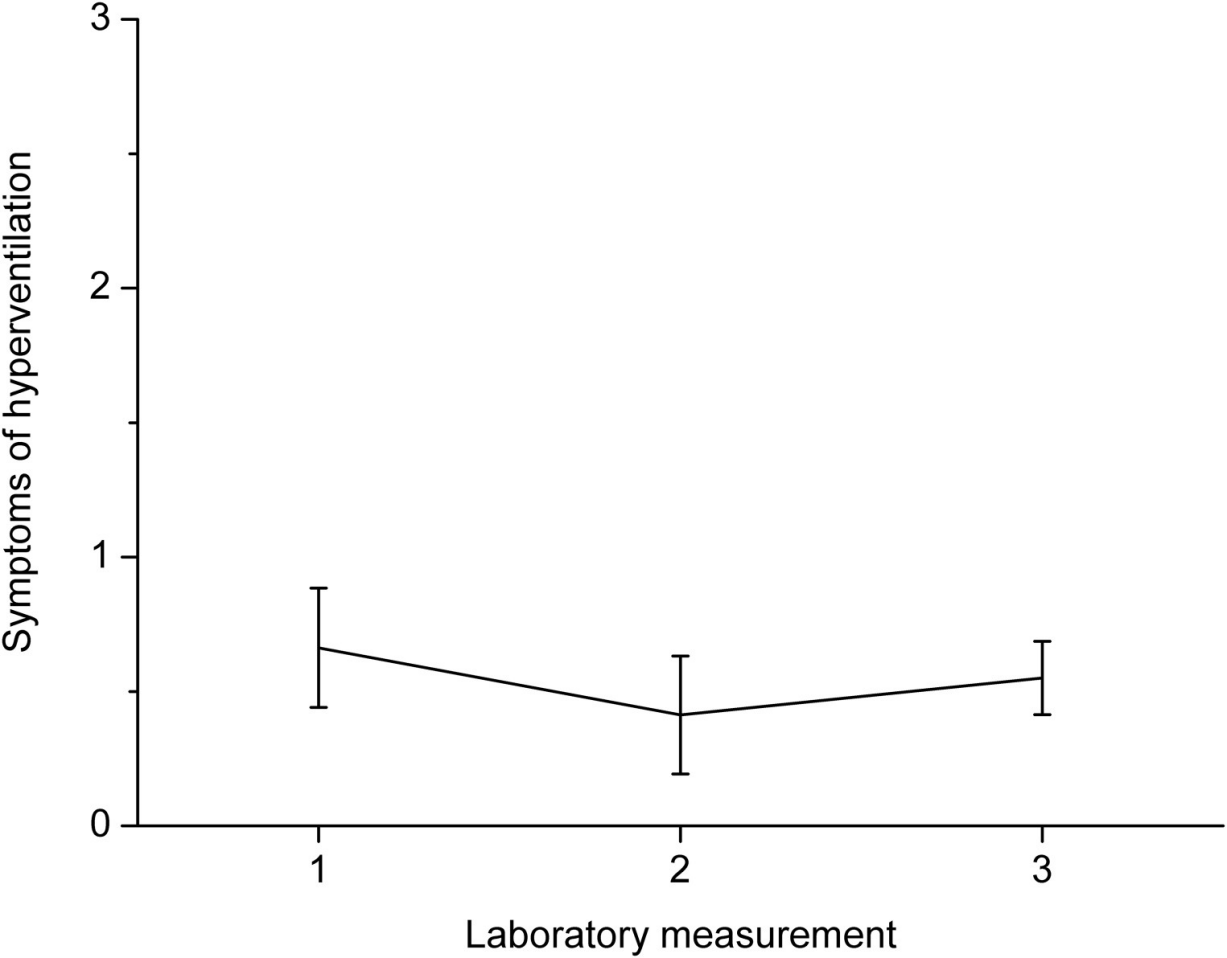
Changes in the intensity of symptoms of hyperventilation between the three laboratory measurements with 95% confidence intervals. Changes computed by subtraction of symptom intensity before paced breathing from symptoms experienced during paced breathing.

**Fig 3:**
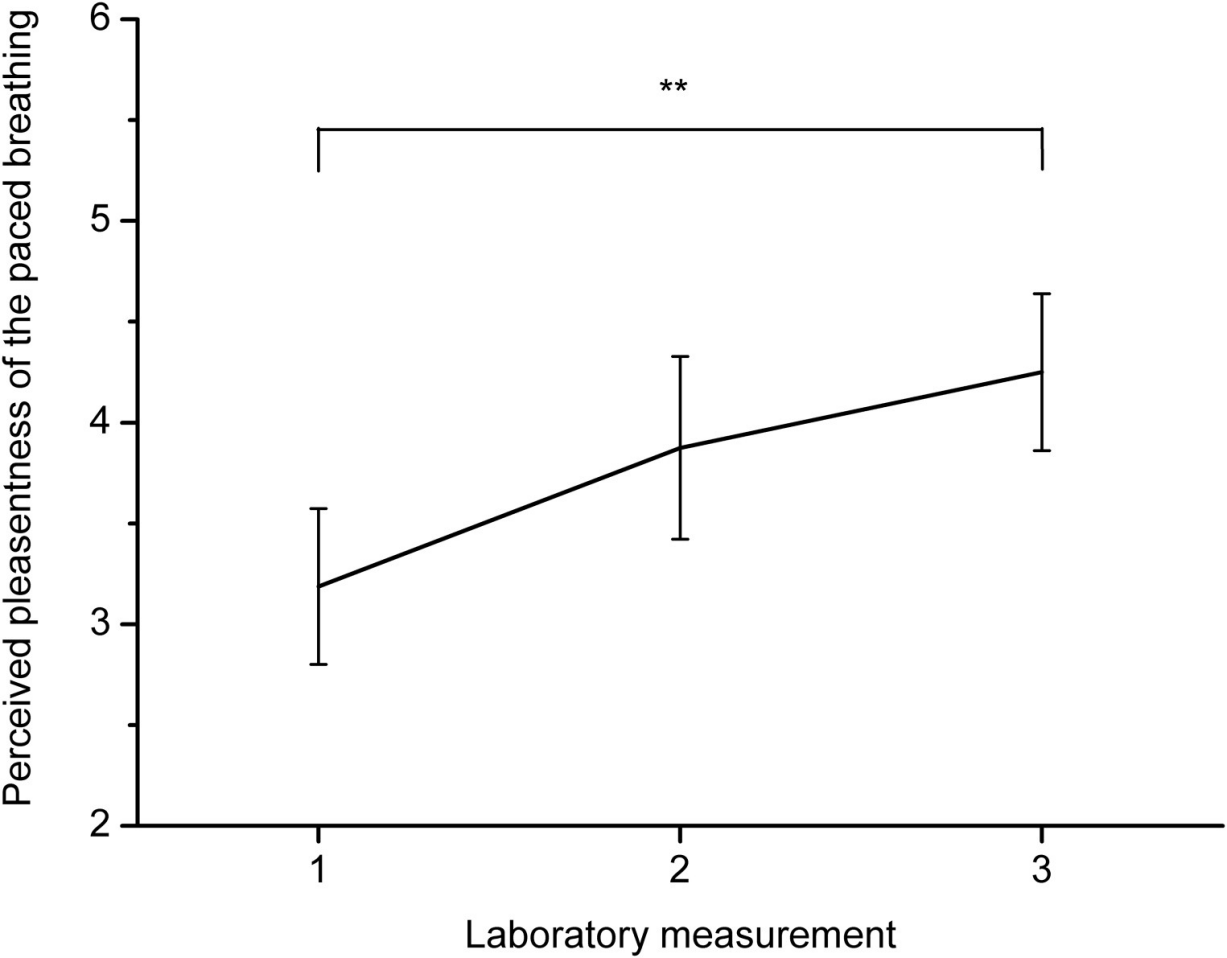
Changes in the perceived pleasantness of the paced breathing task between the three laboratory measurements with 95% confidence intervals. Note. ** *p* < .01; the significance of the difference between the laboratory measurements is indicated.

Participants decreased respiratory rate between baseline and the paced breathing task, *F*(1, 15) = 162.87, *p* < .001, *η*^*2*^ = .92. PetCO_2_ also dropped between baseline and paced breathing, *F*(1, 15) = 14.61, *p* < .01, *η*^*2*^ = .49. Furthermore, PetCO_2_ increased between laboratory measurements, *F*(2, 30) = 4.83, *p* < .05, *η*^*2*^ = .24, and there was an interaction between phase of the experiment (baseline x breathing at 0.1 Hz) and laboratory measurement (first x second x third laboratory session), *F*(2, 30) = 9.22, *p* < .001, *η*^*2*^ = .38. Post-hoc analysis showed that the drop in PetCO_2_ between baseline and paced breathing was significant during the first, *p* < .001, and second laboratory measurements, *p* < .01, but not during the third laboratory measurement, *p* = .17. Changes in PetCO_2_ between baseline and the breathing task for all measurements are presented in Fig 1. Paired sample *t*-tests showed that drop of PetCO_2_ during first and second laboratory measurements did not differ significantly, *t*(15) = 1.63, *p* = .13, but PetCO_2_ dropped less during the third measurement than during the first one, *t*(15) = 4.63, *p* < .001, and the second one, *t*(15) = 2.51, *p* < .05. Thus, results indicate that hyperventilation decreased with training. Data on how many participants experienced a drop in PetCO_2_ below 30 mmHg are presented in Table 1. Analysis of symptoms of hyperventilation showed that they increased between the baseline measurement and measurement after paced breathing, *F*(1, 15) = 5.35, *p* < .05, *η*^*2*^ = .26. However, the intensity of symptoms did not change significantly between laboratory measurements. Changes in symptoms of hyperventilation are presented in Fig 2. Linear regression showed that PetCO_*2*_ during the paced breathing task did not significantly predict symptoms intensity during the first laboratory measurement, *F*(1, 14) = 3.07, *p* = .10, but PetCO_*2*_ predicted hyperventilation symptoms during the second, *F*(1, 14) = 6.30, *p* < .05, and third measurements, *F*(1, 14) = 11.78, *p* < .01 with a *R*^*2*^ of .31 and .46. The perceived pleasantness of the task increased with training, *F*(2, 30) = 5.22, *p* < .05, *η*^*2*^ = .26. Changes in perceived pleasantness of paced breathing are presented in Fig 3. Post-hoc analysis showed that there was a significant difference between pleasantness during the first and third laboratory measurements, *p* < .01.

**Table 1:** Percent of participants with PetCO_2_ below 30 mmHg during baseline and paced breathing for the three days of laboratory measurements.

		Group		
	Measurement	1st	2nd	3rd
PetCO_2_ below 30 mmHg	Baseline	0%	6,3%	6,3%
	Paced breathing	37,5%	12,5%	6,3%

**Fig 4:**
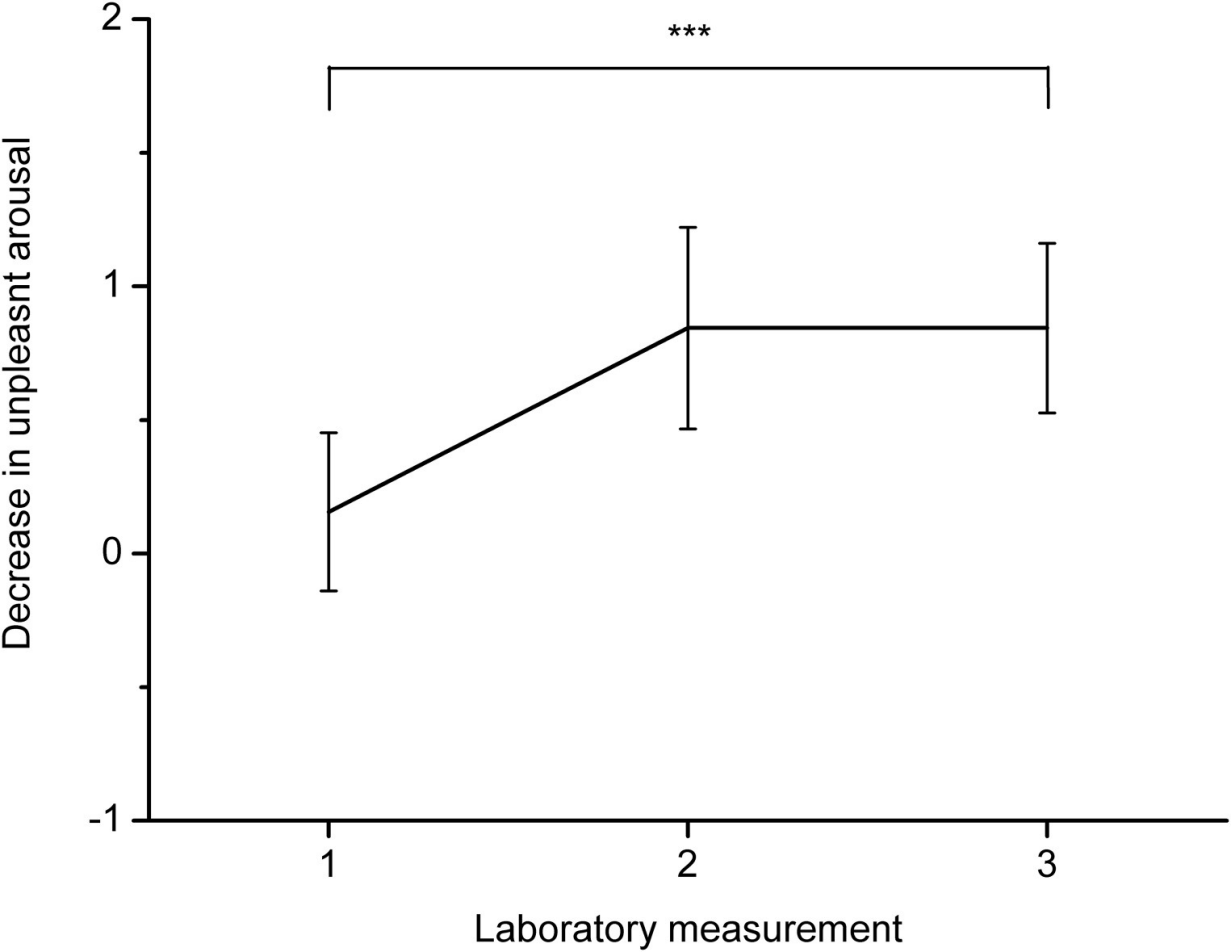
Changes in decrease in unpleasant arousal between the three laboratory measurements with 95% confidence intervals. Changes computed by subtraction of unpleasant arousal after paced breathing from unpleasant arousal during baseline. Note. *** *p* < .001; the significance of the difference between the laboratory measurements is indicated.

The effects of the paced breathing task and the seven days of training on affect were investigated using repeated measures ANOVA. General arousal decreased after the paced breathing task in comparison to baseline measurement, *F*(1, 15) = 32.5, *p* < .001, *η*^*2*^ = .69, but there were no significant changes between laboratory measurements. Valence did not change significantly during the task and between laboratory measurements. Pleasant arousal decreased between baseline and measurement after paced breathing, *F*(1, 15) = 20.16, *p* < .001, *η*^*2*^ = .57, but the observed decrease did not change significantly with training. Unpleasant arousal did not change significantly between baseline and the paced breathing task, but this effect was close to significant, *F*(1, 15) = 4.27, *p* = .056, *η*^*2*^ = .22. Unpleasant arousal decreased more with training, *F*(2, 30) = 4.27, *p* < .05, *η*^*2*^ = .43. Post-hoc comparison showed that the decrease in unpleasant arousal was non-significant during the first laboratory measurement, *p* = .63, but unpleasant arousal decreased during the second, *p* < .05, and third, *p* < .05, laboratory measurements. Thus, the results indicate that training of paced breathing makes it more effective in reducing unpleasant arousal. Changes in the effects of paced breathing on unpleasant arousal between laboratory measurements are presented in Fig 4. Comparisons of changes between baseline and post-task unpleasant arousal scores using paired sample *t*-tests showed that decreases in unpleasant arousal increased between first and second laboratory measurements, *t*(15) = -2.45, *p* < .05, as well as between first and third laboratory measurements, *t*(15) = -3.02, *p* < .01. The mean values for the physiological and self-reported variables are presented in Table 2.

**Table 2:** Means and standard deviations of the physiological and self-reported measures for the different moments of the experiment (baseline and paced breathing) and three laboratory measurements.

	Measurement			
	Moment of measurement	1st	2nd	3rd
PetCO_2_ (mmHg)	Baseline	36.29(3.59)	38.14(3.37)	36.98(3.77)
	During paced breathing	32.06(4.18)	35.14(5.25)	35.78(4.90)
Respiratory Rate (breaths/minute)	Baseline	17.72(3.38)	15.98(3.36)	15.44(3.86)
	During paced breathing	7.05(0.60)	6.90(0.70)	7.05(0.70)
Symptoms of hyperventilation	Baseline	0.26(0.36)	0.21(0.32)	0.25(0.36)
	During paced breathing	0.93(1.18)	0.63(0.83)	0.8(1.36)
Pleasant arousal	Baseline	0.21(1.86)	1.00(1.77)	0.81(2.25)
	After paced breathing	-0.94(2.18)	-0.09(2.09)	-0.34(2.36)
Unpleasant arousal	Baseline	-3.34(1.18)	-2.84(1.39)	-2.72(1.24)
	After paced breathing	-3.50(1.69)	-3.69(1.63)	-3.56(2.05)
Pleasantness of the task		3.19(1.05)	3.87(1.45)	4.25(1.24)

Note. PetCO_2_: pressure of end-tidal carbon dioxide.

## Discussion

The present study investigated the effects of seven days of training paced breathing at 0.1 Hz on adequacy of ventilation during paced breathing and on symptoms of hyperventilation. Furthermore, the present study examined the influence of paced breathing training at 0.1 Hz on breathing-related changes in affective state and perceived pleasantness of the task.

Results showed that the drop in PetCO_2_ during paced breathing at 0.1 Hz was significantly reduced after seven days of training, which indicates improved respiratory homeostasis. A previous study by Vaschillo, Vaschillo, and Lehrer [2] reported cessation of hyperventilation after three days of training, but an anti-hyperventilation instruction was used in this study and the experimenter monitored PetCO_2_ to inform participants when their PetCO_2_ dropped too much. In contrast, the present study used no instruction aimed at altering the depth of breathing. There was a relatively large variation in changes in hyperventilation. For example, the participant who had the largest drop in PetCO_2_ during the first laboratory session (a drop of 7.99 mmHg) still experienced decreases during the second (6.05 mmHg) and third laboratory measurements (7.80 mmHg). However, by the end of the training, the PetCO_2_ of all participants stopped dropping below 30 mmHg, a commonly-used threshold for hyperventilation (except one participant who already had PetCO_2_ below 30 mmHg during baseline; for data on how many participants experienced a drop in PetCO_2_ below 30 mmHg, see Table 1). Thus, the obtained results indicate that training paced breathing at 0.1 Hz significantly reduces the tendency to hyperventilate even when no hyperventilation instruction is used. This finding is important for the use of paced breathing tasks in applied psychophysiology, especially for situations when it is employed without the guidance of a qualified breathing coach, for example in mobile apps which use slow paced breathing as an affect regulation method.

The decrease of hyperventilation observed in the present study may be an effect of increased automatization of the paced breathing task. As a consequence of practise, motor tasks become more automated, which involves changes in neural control and decreased attentional resources required to perform the task [56]. Gradual automatization of paced breathing tasks during training has been shown by Gallego and Perruchet [57]. In their study, eight days of training of paced breathing gradually decreased the attentional resources used to perform paced breathing. Presumably, partial automatization of paced breathing during training alters neural control of breathing, allowing an increase in the role of the CO_2_ homeostatic mechanism in the control of breathing. The decrease of hyperventilation could be also the result of automatic optimization of breathing aimed at reducing respiratory work. Hyperventilation requires increased tidal volume, which already must be augmented during slow breathing. Therefore, hyperventilation is costly in terms of the elastic work of breathing [58]. It is thought that the minimization of respiratory work is one of the main factors regulating breathing [59, 60]. Therefore, training should lead to gradual optimization of breathing, which results in decreased hyperventilation.

Participants reported a small increase in symptoms of hyperventilation during breathing at 0.1 Hz. In line with expectations, the results suggest that the intensity of symptoms was related to magnitude of hyperventilation. However, despite the decrease in the drop of PetCO_2_, the intensity of symptoms did not decrease significantly with training. This suggests that even after training some participants experienced large enough disruption of arterial CO_2_ to produce noticeable symptoms. Despite the fact that reported hyperventilation symptoms can be considered minor in most participants, two participants reported increases of symptoms by 2.8 and 3.4 on a 7 point scale while experiencing a drop of PetCO_2_ by 4.86 and 5.53 mmHg, respectively; several participants experienced a comparable drop of PetCO_2_ with increases in symptom intensity smaller than 1. This result suggests the existence of significant individual differences in sensitivity to physiological changes produced by the disruption of arterial CO_2_ homeostasis. Individual differences in symptom intensity during voluntary hyperventilation have previously been shown to be related to trait anxiety and anxiety sensitivity [61]. If such psychological traits affect the experience of symptoms during slow paced breathing, hyperventilation may be of particular importance among the population with higher levels of anxiety.

The effects of paced breathing tasks on affective state changed with training. It was hypothesised that training paced breathing is necessary to produce consistent anti-arousal changes in affective state. The results support this hypothesis because the effects of paced breathing on unpleasant arousal appeared during the second and third laboratory measurements and not during the first. Unpleasant arousal is a dimension of affective state that extends from states of high arousal and negative valence (e.g. irritation and nervousness) to states of low arousal and positive valence (e.g. relaxation and calmness [47]). The task was also perceived as more pleasurable with training. Thus, the present study suggests that training increases the relaxing effects of paced breathing at 0.1 Hz and makes it more pleasurable. Therefore, the lack of effects of paced breathing on affect reported by some previous studies could be caused by the participants’ lack of experience with paced breathing [44, 45, 46, 27, 28].

Multiple mechanisms may be responsible for the improvement of the affective effects of paced breathing with training. Studies have shown that slow paced breathing increases breathing discomfort (dyspnea; [62, 63, 64]). Mitigation of dyspnea is considered one of the factors determining breathing behavior [65]. Training may allow the optimization of breathing mechanics to decrease dyspnea. The observed increase in pleasantness of the task suggests a decrease of breathing discomfort with training. Changes in affective state may also be a result of the partial automatization of the task. The shift from controlled processing to automatic processes during motor skill acquisition is typically associated with reduced mental effort required to perform a task [66]. This may improve the relaxing properties of slow paced breathing.

The results of the present study has consequences for the generalization of the results of previous studies and for future research on the effects of slow paced breathing. Previous research conducted on untrained individuals presumably failed to show some effects of paced breathing exercises that are experienced by those who perform such exercises regularly. Therefore, future research which aims to examine the effects of slow paced breathing should employ training.

The present study also has consequences for the use of paced breathing as an applied method. Some methods that use slow paced breathing employ anti-hyperventilation instructions [2, 35]. However, the issue of hyperventilation is often overlooked and no anti-hyperventilation instructions are used [17, 33, 38]. Furthermore, paced breathing is currently widely implemented in many mobile applications [20, 21]. For this reason, it is certainly widely used without the assistance of a breathing coach, which increases the risk of hyperventilation. The present study suggests that there exists a relatively large variation in changes in hyperventilation during paced breathing training. Because of individuals who still experience significant drops of PetCO_2_ after the training, methods to avoid hyperventilation—such as anti-hyperventilation instructions [28] or, when possible, capnometer biofeedback [36]—should be routinely included as part of paced breathing training and implemented in mobile applications. The present and previous studies suggest that symptoms of hyperventilation can be considered a mild adverse effect during slow paced breathing training. Therefore, effective avoidance of hyperventilation may increase tolerability and adherence to breathing training.

The issue of hyperventilation during paced breathing is of particular importance among chronic hyperventilators. Around 6–10% of the population tend to hyperventilate chronically [67]. This subpopulation can be particularly prone to look for some form of breathing training because of hyperventilation-related dyspnea. Due to low resting arterial CO_2_ level, paced breathing without paying attention to hyperventilation may lead to deep hyperventilation with severe symptoms. For the safety of paced breathing training it is important to investigate the effects of paced breathing on hyperventilation in this group.

To sum up, the present study showed that during breathing at 0.1 Hz, even without any anti-hyperventilation instruction, the drop in PetCO_2_ decreases with training. However, there was relatively high variability, which indicates that even after training some participants experience a significant drop in PetCO_2_. Paced breathing produced mostly minor increases in hyperventilation symptoms, however some subjects experienced symptoms of medium intensity. Symptom intensity did not change as a result of training. Furthermore, these results indicate that the affective effects of paced breathing improve with training, making paced breathing more relaxing. Unpleasant arousal did not change during the first performance of paced breathing, but it begin to decrease during the second measurement (third day of training). Thus, the present results suggest that both CO_2_ homeostasis and the affective effects of paced breathing improve with training.

## References

[1] Lehrer PM, Vaschillo E, Vaschillo B. Resonant frequency biofeedback training to increase cardiac variability: Rationale and manual for training. Applied Psychophysiology and Biofeedback; 2000;25(3):177–91. 10.1023/a:1009554825745 10999236

[2] Vaschillo EG, Vaschillo B, Lehrer PM. Characteristics of Resonance in Heart Rate Variability Stimulated by Biofeedback. Applied Psychophysiology and Biofeedback. Springer Nature; 2006 6;31(2):129–42. 10.1007/s10484-006-9009-3 16838124

[3] Wheat AL, Larkin KT. Biofeedback of Heart Rate Variability and Related Physiology: A Critical Review. Applied Psychophysiology and Biofeedback; 2010 5 5;35(3):229–42. 10.1007/s10484-010-9133-y 20443135

[4] Karavidas MK, Lehrer PM, Vaschillo E, Vaschillo B, Marin H, Buyske S, et al Preliminary Results of an Open Label Study of Heart Rate Variability Biofeedback for the Treatment of Major Depression. Applied Psychophysiology and Biofeedback; 2007 3 1;32(1):19–30. 10.1007/s10484-006-9029-z 17333315

[5] Siepmann M, Aykac V, Unterdörfer J, Petrowski K, Mueck-Weymann M. A Pilot Study on the Effects of Heart Rate Variability Biofeedback in Patients with Depression and in Healthy Subjects. Applied Psychophysiology and Biofeedback; 2008 9 19;33(4):195–201. 10.1007/s10484-008-9064-z 18807175

[6] Goessl VC, Curtiss JE, Hofmann SG. The effect of heart rate variability biofeedback training on stress and anxiety: a meta-analysis. Psychological Medicine [Internet]. Cambridge University Press (CUP); 2017 5 8;47(15):2578–86. 10.1017/S0033291717001003 28478782

[7] Lehrer PM, Vaschillo E, Vaschillo B, Lu S-E, Scardella A, Siddique M, et al Biofeedback Treatment for Asthma. Chest; 2004 8;126(2):352–61. 10.1378/chest.126.2.352 15302717

[8] Hassett AL, Radvanski DC, Vaschillo EG, Vaschillo B, Sigal LH, Karavidas MK, et al A Pilot Study of the Efficacy of Heart Rate Variability (HRV) Biofeedback in Patients with Fibromyalgia. Applied Psychophysiology and Biofeedback; 2007 1 12;32(1):1–10. 10.1007/s10484-006-9028-0 17219062

[9] Tan G, Dao TK, Farmer L, Sutherland RJ, Gevirtz R. Heart Rate Variability (HRV) and Posttraumatic Stress Disorder (PTSD): A Pilot Study. Applied Psychophysiology and Biofeedback; 2010 8 3;36(1):27–35. 10.1007/s10484-010-9141-y 20680439

[10] Zucker TL, Samuelson KW, Muench F, Greenberg MA, Gevirtz RN. The Effects of Respiratory Sinus Arrhythmia Biofeedback on Heart Rate Variability and Posttraumatic Stress Disorder Symptoms: A Pilot Study. Applied Psychophysiology and Biofeedback; 2009 4 25;34(2):135–43. 10.1007/s10484-009-9085-2 19396540

[11] Chen S, Sun P, Wang S, Lin G, Wang T. Effects of heart rate variability biofeedback on cardiovascular responses and autonomic sympathovagal modulation following stressor tasks in prehypertensives. Journal of Human Hypertension. 2015 4 30;30(2):105–11. 10.1038/jhh.2015.27 25924910

[12] Lin G, Xiang Q, Fu X, Wang S, Wang S, Chen S, et al Heart Rate Variability Biofeedback Decreases Blood Pressure in Prehypertensive Subjects by Improving Autonomic Function and Baroreflex. The Journal of Alternative and Complementary Medicine; 2012 2;18(2):143–52. 10.1089/acm.2010.0607 22339103

[13] Zou Y, Zhao X, Hou Y-Y, Liu T, Wu Q, Huang Y-H, et al Meta-Analysis of Effects of Voluntary Slow Breathing Exercises for Control of Heart Rate and Blood Pressure in Patients With Cardiovascular Diseases. The American Journal of Cardiology; 2017 7;120(1):148–53. 10.1016/j.amjcard.2017.03.247 28502461

[14] Chalaye P, Goffaux P, Lafrenaye S, Marchand S. Respiratory Effects on Experimental Heat Pain and Cardiac Activity. Pain Medicine; 2009 11;10(8):1334–40. 10.1111/j.1526-4637.2009.00681.x 19671085

[15] Jafari H, Courtois I, Van den Bergh O, Vlaeyen JWS, Van Diest I. Pain and respiration. PAIN; 2017 6;158(6):995–1006. 10.1097/j.pain.0000000000000865 28240995

[16] Zautra AJ, Fasman R, Davis MC, Craig AD (Bud). The effects of slow breathing on affective responses to pain stimuli: An experimental study. Pain; 2010 4;149(1):12–8. 10.1016/j.pain.2009.10.001 20079569

[17] Laborde S, Hosang T, Mosley E, Dosseville F. Influence of a 30-Day Slow-Paced Breathing Intervention Compared to Social Media Use on Subjective Sleep Quality and Cardiac Vagal Activity. Journal of Clinical Medicine; 2019 2 6;8(2):193 10.3390/jcm8020193 30736268 PMC6406675

[18] Tsai HJ, Kuo TBJ, Lee G-S, Yang CCH. Efficacy of paced breathing for insomnia: Enhances vagal activity and improves sleep quality. Psychophysiology; 2014 9 19;52(3):388–96. 10.1111/psyp.12333 25234581

[19] Zaccaro A, Piarulli A, Laurino M, Garbella E, Menicucci D, Neri B, et al How Breath-Control Can Change Your Life: A Systematic Review on Psycho-Physiological Correlates of Slow Breathing. Frontiers in Human Neuroscience; 2018 9 7;12 10.3389/fnhum.2018.00353 30245619 PMC6137615

[20] Christmann CA, Hoffmann A, Bleser G. Stress Management Apps With Regard to Emotion-Focused Coping and Behavior Change Techniques: A Content Analysis. JMIR mHealth and uHealth; 2017 2 23;5(2):e22 10.2196/mhealth.6471 28232299 PMC5344985

[21] Coulon SM, Monroe CM, West DS. A Systematic, Multi-domain Review of Mobile Smartphone Apps for Evidence-Based Stress Management. American Journal of Preventive Medicine; 2016 7;51(1):95–105. 10.1016/j.amepre.2016.01.026 26993534

[22] Del Negro CA, Funk GD, Feldman JL. Breathing matters. Nature Reviews Neuroscience; 2018 5 8;19(6):351–67. 10.1038/s41583-018-0003-6 29740175 PMC6636643

[23] Homma I, Masaoka Y. Breathing rhythms and emotions. Experimental Physiology; 2008 8 14;93(9):1011–21. 10.1113/expphysiol.2008.042424 18487316

[24] Colebatch JG, Adams L, Murphy K, Martin AJ, Lammertsma AA, Tochon-Danguy HJ, et al Regional cerebral blood flow during volitional breathing in man. The Journal of Physiology; 1991 11 1;443(1):91–103. 10.1113/jphysiol.1991.sp018824 1822545 PMC1179832

[25] McKay LC, Evans KC, Frackowiak RSJ, Corfield DR. Neural correlates of voluntary breathing in humans. Journal of Applied Physiology; 2003 9;95(3):1170–8. 10.1152/japplphysiol.00641.2002 12754178

[26] Anderson DE, McNeely JD, Windham BG. Device-guided slow-breathing effects on end-tidal CO2and heart-rate variability. Psychology, Health & Medicine; 2009 12;14(6):667–79. 10.1080/13548500903322791 PMC4054864 20183539

[27] Szulczewski MT, Rynkiewicz A. The effects of breathing at a frequency of 0.1 Hz on affective state, the cardiovascular system, and adequacy of ventilation. Psychophysiology; 2018 7 16;55(12):e13221 10.1111/psyp.13221 30010195

[28] Szulczewski MT, An Anti-hyperventilation Instruction Decreases the Drop in End-tidal CO_2_ and Symptoms of Hyperventilation During Breathing at 0.1 Hz. Applied Psychophysiology and Biofeedback; Forthcoming 2019 10.1007/s10484-019-09438-y 31119404 PMC6686033

[29] Carter MM, Suchday S, Gore KL. The utility of the ASI factors in predicting response to voluntary hyperventilation among nonclinical participants. Journal of Anxiety Disorders; 2001 5;15(3):217–30. 10.1016/s0887-6185(01)00061-5 11442140

[30] Hornsveld H, Garssen B, Dop MF, van Spiegel P. Symptom reporting during voluntary hyperventilation and mental load: Implications for diagnosing hyperventilation syndrome. Journal of Psychosomatic Research; 1990 1;34(6):687–97. 10.1016/0022-3999(90)90113-i 2290141

[31] Thyer BA, Papsdorf JD, Wright P. Physiological and psychological effects of acute intentional hyperventilation. Behaviour Research and Therapy; 1984;22(5):587–90. 10.1016/0005-7967(84)90063-9 6508709

[32] Van de Borne P, Mezzetti S, Montano N, Narkiewicz K, Degaute JP, Somers VK. Hyperventilation alters arterial baroreflex control of heart rate and muscle sympathetic nerve activity. American Journal of Physiology-Heart and Circulatory Physiology; 2000 8;279(2):H536–H541. 10.1152/ajpheart.2000.279.2.H536 10924051

[33] Sood R, Sood A, Wolf SL, Linquist BM, Liu H, Sloan JA, et al Paced breathing compared with usual breathing for hot flashes. Menopause: The Journal of The North American Menopause Society; 2012 9;1 10.1097/gme.0b013e31826934b6 22990758

[34] Kim S, Wollburg E, Roth WT. Opposing Breathing Therapies for Panic Disorder. The Journal of Clinical Psychiatry; 2012 7 15;73(07):931–9. 10.4088/jcp.11m07068 22901344

[35] Meuret AE, Ritz T, Wilhelm FH, Roth WT, Rosenfield D. Hypoventilation Therapy Alleviates Panic by Repeated Induction of Dyspnea. Biological Psychiatry: Cognitive Neuroscience and Neuroimaging. Elsevier BV; 2018 6;3(6):539–45. 10.1016/j.bpsc.2018.01.010 29573981 PMC6019126

[36] Meuret AE, Wilhelm FH, Ritz T, Roth WT. Feedback of end-tidal pCO2 as a therapeutic approach for panic disorder. Journal of Psychiatric Research; 2008 6;42(7):560–8. 10.1016/j.jpsychires.2007.06.005 17681544 PMC2890048

[37] Ritz T, Rosenfield D, Steele AM, Millard MW, Meuret AE. Controlling Asthma by Training of Capnometry-Assisted Hypoventilation (CATCH) vs Slow Breathing. Chest; 2014 11;146(5):1237–47.: 10.1378/chest.14-0665 25122497 PMC4219339

[38] Laborde S, Allen MS, Göhring N, Dosseville F. The effect of slow-paced breathing on stress management in adolescents with intellectual disability. Journal of Intellectual Disability Research. Wiley; 2016 12 9;61(6):560–7. 10.1111/jir.12350 27933677

[39] Prinsloo GE, Derman WE, Lambert MI, Rauch HGL. The effect of a single episode of short duration heart rate variability biofeedback on measures of anxiety and relaxation states. International Journal of Stress Management; 2013;20(4):391–411. 10.1037/a0034777

[40] Sherlin L, Gevirtz R, Wyckoff S, Muench F. Effects of respiratory sinus arrhythmia biofeedback versus passive biofeedback control. International Journal of Stress Management; 2009;16(3):233–48. 10.1037/a0016047

[41] Wells R, Outhred T, Heathers JAJ, Quintana DS, Kemp AH. Matter Over Mind: A Randomised-Controlled Trial of Single-Session Biofeedback Training on Performance Anxiety and Heart Rate Variability in Musicians. PLoS ONE; 2012 10 4;7(10):e46597 10.1371/journal.pone.0046597 23056361 PMC3464298

[42] Lin IM, Tai LY, Fan SY. Breathing at a rate of 5.5breaths per minute with equal inhalation-to-exhalation ratio increases heart rate variability. International Journal of Psychophysiology; 2014 3;91(3):206–11. 10.1016/j.ijpsycho.2013.12.006 24380741

[43] Van Diest I, Verstappen K, Aubert AE, Widjaja D, Vansteenwegen D, Vlemincx E. Inhalation/Exhalation Ratio Modulates the Effect of Slow Breathing on Heart Rate Variability and Relaxation. Applied Psychophysiology and Biofeedback; 2014 8 26;39(3–4):171–80. 10.1007/s10484-014-9253-x 25156003

[44] Cappo BM, Holmes DS. The utility of prolonged respiratory exhalation for reducing physiological and psychological arousal in non-threatening and threatening situations. Journal of Psychosomatic Research; 1984 1;28(4):265–73. 10.1016/0022-3999(84)90048-5 6481661

[45] Stark R, Schienle A, Walter B, Vaitl D. Effects of paced respiration on heart period and heart period variability. Psychophysiology; 2000 5;37(3):302–9. 10.1111/1469-8986.3730302 10860408

[46] Steffen PR, Austin T, DeBarros A, Brown T. The Impact of Resonance Frequency Breathing on Measures of Heart Rate Variability, Blood Pressure, and Mood. Frontiers in Public Health; 2017 8 25;5 10.3389/fpubh.2017.00222 28890890 PMC5575449

[47] Sakairi Y, Nakatsuka K, Shimizu T. Development of the Two-Dimensional Mood Scale for self-monitoring and self-regulation of momentary mood states. Japanese Psychological Research; 2013 6 5;n/a–n/a. 10.1111/jpr.12021

[48] Barrett LF, Mesquita B, Ochsner KN, Gross JJ. The Experience of Emotion. Annual Review of Psychology; 2007 1;58(1):373–403. 10.1146/annurev.psych.58.110405.085709 17002554 PMC1934613

[49] Russell JA. Core affect and the psychological construction of emotion. Psychological Review; 2003;110(1):145–72. 10.1037/0033-295x.110.1.145 12529060

[50] Yik M, Russell JA, Steiger JH. A 12-point circumplex structure of core affect. Emotion; 2011;11(4):705–31. 10.1037/a0023980 21707162

[51] Yik MS, Russell JA, Barrett LF. Structure of self-reported current affect: Integration and beyond. Journal of Personality and Social Psychology; 1999;77(3):600–19. 10.1037/0022-3514.77.3.600

[52] Van Dixhoorn J, Duivenvoorden HJ. Efficacy of Nijmegen questionnaire in recognition of the hyperventilation syndrome. Journal of Psychosomatic Research; 1985 1;29(2):199–206. 10.1016/0022-3999(85)90042-x 4009520

[53] Mathôt S, Schreij D, Theeuwes J. OpenSesame: An open-source, graphical experiment builder for the social sciences. Behavior Research Methods; 2011 11 16;44(2):314–24. 10.3758/s13428-011-0168-7 22083660 PMC3356517

[54] Wang Y-P, Kuo TBJ, Lai C-T, Chu J-W, Yang CCH. Effects of respiratory time ratio on heart rate variability and spontaneous baroreflex sensitivity. Journal of Applied Physiology; 2013 12;115(11):1648–55. 10.1152/japplphysiol.00163.2013 24092689

[55] Morey RD. Confidence Intervals from Normalized Data: A correction to Cousineau (2005). Tutorials in Quantitative Methods for Psychology; 2008 9 1;4(2):61–4. 10.20982/tqmp.04.2.p061

[56] Doyon J, Bellec P, Amsel R, Penhune V, Monchi O, Carrier J, et al Contributions of the basal ganglia and functionally related brain structures to motor learning. Behavioural Brain Research; 2009 4;199(1):61–75. 10.1016/j.bbr.2008.11.012 19061920

[57] Gallego J, Perruchet P. Effect of practice on the voluntary control of a learned breathing pattern. Physiology & Behavior; 1991 2;49(2):315–9. 10.1016/0031-9384(91)90049-t 2062903

[58] Otis AB. The Work of Breathing. Physiological Reviews; 1954 7;34(3):449–58. 10.1152/physrev.1954.34.3.449 13185751

[59] Mortola JP. How to breathe? Respiratory mechanics and breathing pattern. Respiratory Physiology & Neurobiology; 2019 3;261:48–54. 10.1016/j.resp.2018.12.005 30605732

[60] Poon CS. Introduction: Optimization Hypothesis in the Control of Breathing. Control of Breathing and Its Modeling Perspective; 1992;371–84. 10.1007/978-1-4757-9847-0_66

[61] Rapee RM, Medoro L. Fear of physical sensations and trait anxiety as mediators of the response to hyperventilation in nonclinical subjects. Journal of Abnormal Psychology; 1994;103(4):693–9. 10.1037/0021-843x.103.4.693 7822570

[62] Allen B, Friedman BH. Positive emotion reduces dyspnea during slow paced breathing. Psychophysiology; 2012 1 31;49(5):690–6. 10.1111/j.1469-8986.2011.01344.x 22292794

[63] Chonan T, Mulholland MB, Altose MD, Cherniack NS. Effects of changes in level and pattern of breathing on the sensation of dyspnea. Journal of Applied Physiology [Internet]. American Physiological Society; 1990 10;69(4):1290–5. Available from: 10.1152/jappl.1990.69.4.1290 2262446

[64] Denot-Ledunois S, Vardon G, Gallego J. 26. Effects of voluntary changes in breathing frequency on respiratory comfort. Biological Psychology; 1996 1;43(3):257 10.1016/0301-0511(96)88251-2 9792485

[65] Oku Y, Saidel GM, Altose MD, Cherniack NS. Perceptual contributions to optimization of breathing. Annals of Biomedical Engineering; 1993 9;21(5):509–15. 10.1007/bf02584333 8239091

[66] Shenhav A, Musslick S, Lieder F, Kool W, Griffiths TL, Cohen JD, et al Toward a Rational and Mechanistic Account of Mental Effort. Annual Review of Neuroscience; 2017 7 25;40(1):99–124. 10.1146/annurev-neuro-072116-031526 28375769

[67] Boulding R, Stacey R, Niven R, Fowler SJ. Dysfunctional breathing: a review of the literature and proposal for classification. European Respiratory Review; 2016 8 31;25(141):287–94. 10.1183/16000617.0088-2015 27581828 PMC9487208

